# Impact of the Secretome of Human Mesenchymal Stem Cells on Brain Structure and Animal Behavior in a Rat Model of Parkinson's Disease

**DOI:** 10.5966/sctm.2016-0071

**Published:** 2016-09-22

**Authors:** Fábio G. Teixeira, Miguel M. Carvalho, Krishna M. Panchalingam, Ana J. Rodrigues, Bárbara Mendes‐Pinheiro, Sandra Anjo, Bruno Manadas, Leo A. Behie, Nuno Sousa, António J. Salgado

**Affiliations:** ^1^ Life and Health Sciences Research Institute, School of Health Sciences, University of Minho, Braga, Portugal; ^2^ ICVS/3B's ‐ PT Government Associate Laboratory, Braga/Guimarães, Portugal; ^3^ Pharmaceutical Production Research Facility, Department of Chemical and Petroleum Engineering, University of Calgary, Calgary, Alberta, Canada; ^4^ Center for Neuroscience and Cell Biology, University of Coimbra, Coimbra, Portugal; ^5^ Faculty of Sciences and Technology, University of Coimbra, Coimbra, Portugal; ^6^ Biocant ‐ Biotechnology Innovation Center, Cantanhede, Portugal

**Keywords:** Mesenchymal stem cells, Parkinson's disease, Secretome, Dopaminergic neurons, Neuroprotection

## Abstract

Research in the last decade strongly suggests that mesenchymal stem cell (MSC)‐mediated therapeutic benefits are mainly due to their secretome, which has been proposed as a possible therapeutic tool for the treatment of Parkinson's disease (PD). Indeed, it has been shown that the MSC secretome increases neurogenesis and cell survival, and has numerous neuroprotective actions under different conditions. Additionally, using dynamic culturing conditions (through computer‐controlled bioreactors) can further modulate the MSC secretome, thereby generating a more potent neurotrophic factor cocktail (i.e., conditioned medium). In this study, we have characterized the MSC secretome by proteomic‐based analysis, investigating its therapeutic effects on the physiological recovery of a 6‐hydroxidopamine (6‐OHDA) PD rat model. For this purpose, we injected MSC secretome into the substantia nigra (SNc) and striatum (STR), characterizing the behavioral performance and determining histological parameters for injected animals versus untreated groups. We observed that the secretome potentiated the increase of dopaminergic neurons (i.e., tyrosine hydroxylase‐positive cells) and neuronal terminals in the SNc and STR, respectively, thereby supporting the recovery observed in the Parkinsonian rats’ motor performance outcomes (assessed by rotarod and staircase tests). Finally, proteomic characterization of the MSC secretome (through combined mass spectrometry analysis and Bioplex assays) revealed the presence of important neuroregulatory molecules, namely cystatin C, glia‐derived nexin, galectin‐1, pigment epithelium‐derived factor, vascular endothelial growth factor, brain‐derived neurotrophic factor, interleukin‐6, and glial cell line‐derived neurotrophic factor. Overall, we concluded that the use of human MSC secretome alone was able to partially revert the motor phenotype and the neuronal structure of 6‐OHDA PD animals. This indicates that the human MSC secretome could represent a novel therapeutic for the treatment of PD. Stem Cells Translational Medicine *2017;6:634–646*

**Significance Statement**

It has been suggested that the therapeutic effects of human mesenchymal stem cells (hMSCs) in central nervous system (CNS) regenerative medicine are mediated by the active secretion of bioactive molecules, which is known as the secretome. This study demonstrated that the injection of hMSC secretome in a 6‐hydroxidopamine Parkinson's disease (PD) rat model was able to revert the Parkinsonian phenotype, potentiating the recovery of dopaminergic neurons in both the substantia nigra and striatum, thereby supporting the motor recovery observed in the PD rats. This work shows the modulatory role of the hMSC secretome in brain repair, further indicating that such cell‐free therapies could represent the basis of future strategies for the treatment of PD.

## Introduction

Parkinson's disease (PD), the second most prevalent neurodegenerative disorder worldwide, is clinically characterized by a progressive degeneration of dopaminergic (DA) neurons in several dopaminergic networks 1, 2. This process is most intensively observed in the mesostriatal pathway at the level of the substantia nigra pars compacta (SNc) 1, 2, 3, 4. As a result of this neuronal loss, patients develop several motor complications, including rigidity, bradykinesia, and postural instability 5. The use of levodopa (l‐DOPA) has been considered as the standard treatment for PD and for the reduction of its major symptoms 6, 7. Additionally, in conjunction with l‐DOPA treatment, the use of dopamine reuptake inhibitors, DA agonists, and muscarinic antagonists also have positive clinical effects 6, 8. However, despite these pharmacological advances, most of these treatments have been shown to be insufficient, presenting some undesirable side effects, long‐term inefficiency, and the inability to recover lost DA neurons or to protect the viability of the remaining ones 9, 10, 11, 12, 13. Surgical treatments, such as deep‐brain stimulation, have been applied as an alternative in patients in whom pharmacological treatment is no longer effective 11, 14, 15. However, as with drug treatments, the apparent clinical recovery after surgery does not last and the progression of the degenerative process is not avoided 16, 17.

The use of a human mesenchymal stem cell (hMSC)‐based strategy has emerged as a potential alternative therapy for PD 18, 19, 20. In fact, several studies in PD animal models have already shown that the transplantation of hMSCs acts as a promoter of neuroprotection and/or neural function 21, 22, 23. For instance, Venkataramana et al. 24 showed that transplanted hMSCs, which survived transplantation, integrated into the brain parenchyma and migrated toward the ipsilateral nigra. Whereas some reports propose that the differentiation of hMSCs into DA neurons or neural lineages is the principal outcome for hMSC‐mediated PD recovery, others indicate that this functional recovery is promoted by the hMSC secretome 25, 26. Indeed, the secretome hypothesis has stimulated a distinctive perspective for the use of hMSCs as a potential cell‐free therapeutic tool for central nervous system (CNS) regeneration 25, 27. The role that the hMSC secretome (i.e., conditioned medium [CM]) plays in vivo has been investigated in several studies that have shown that transplanted hMSCs (from different tissue sources) were able to secrete important trophic factors such as epidermal growth factor, vascular endothelial growth factor (VEGF), neurotrophin‐3, fibroblast growth factor 2 (FGF‐2), hepatocyte growth factor (HGF), brain‐derived neurotrophic factor (BDNF), stromal‐derived factor 1, glial cell line‐derived neurotrophic factor (GDNF), and fibroblast growth factor 20 22, 28, 29, 30, 31, 32, 33. Moreover, we have recently shown that the administration of a single injection of the hMSC secretome into the dentate gyrus of rat hippocampus, with no further cell transplantation, was able to increase endogenous cell proliferation and neuronal cell densities, while simultaneously increasing the tissue levels of FGF‐2 34. These results, per se, indicate that the secretome itself could be a possible therapeutic tool for CNS regenerative medicine, and specifically for PD. Therefore, in the present work, we analyzed the effects of the hMSC secretome on the DA neuronal cell survival and motor function in an animal model of PD, as well as identified and characterized possible new neuroregulatory molecules that mediate these actions.

## Methods

### Expansion of hMSCs in Stirred Computer‐Controlled Bioreactors and Collection of the Secretome

We have observed that the dynamic culture of hMSCs in computer‐controlled suspension bioreactors enriched the neuroregulatory properties of their secretome 35. In this study, we collected the secretome of hMSCs that were cultured in dynamic environments.

### Preparation of Suspension Bioreactors

A DASGIP Parallel Bioreactor system (Eppendorf, Jülich, Germany, https://www.eppendorf.com) was used for the expansion of hMSCs. Before inoculating hMSCs into the DASGIP bioreactors, the 500‐ml suspension bioreactors and modified Teflon four‐paddle impellers (Pharmaceutical Production Research Facility, University of Calgary, Calgary, ON, Canada, http://pprf.ca/) were siliconized using Sigmacote (Sigma‐Aldrich, St. Louis, MO, http://www.sigmaaldrich.com) to minimize cell attachment to the sides of the bioreactor vessel and the impeller. After siliconization and autoclaving of the vessels, the DASGIP system was set up and calibrated according to protocols provided by the manufacturer. The bioreactors were maintained at 37°C, using a heating jacket; 21% oxygen in the headspace; pH of 7.4, controlled by a gas mixture connected to oxygen, nitrogen, carbon dioxide, and air tanks; and agitated at 52 rpm using a magnetic stir plate under the bioreactors.

### Preparation of Microcarriers, Inoculation of hMSCs, and CM Collection

Cytodex 3 microcarriers (GE Healthcare Biosciences, Pittsburgh, PA, http://www.gelifesciences.com) were used for this study and were prepared as previously described 35. hMSCs derived from bone marrow were expanded in static culture (as described in Teixeira et al. 35) for two passages before inoculation into the DASGIP bioreactors. Cells were then harvested and inoculated into the bioreactors at a density of 24,000 cells per milliliter (based on the final volume of 500 ml) and the volume of the medium in bioreactors was maintained at 325 ml for the first 24 hours to increase cell attachment. After 24 hours, culture volume was increased to 500 ml to bring the final microcarrier density to 2.0 g/l. Cells were cultured on the microcarriers for 72 hours, then the bioreactors were removed from the DASGIP system and placed in a biosafety cabinet for 10 minutes to allow the microcarriers to settle. The supernatant was removed from the bioreactors, and the microcarriers were washed once with 100 ml of Neurobasal‐A medium (Thermo Fisher Scientific Life Sciences, Waltham, MA, http://www.thermofisher.com). Following this, 500 ml of Neurobasal‐A medium with 1% of kanamycin (Thermo Fisher Scientific Life Sciences) was added to the bioreactors and the bioreactors were placed back into the DASGIP control system for 24 hours. After 24 hours, the bioreactors were again removed, placed in a biosafety cabinet for 10 minutes to allow the microcarriers to settle, and then the supernatant was harvested and centrifuged at 300*g* for 10 minutes to remove any cell debris. This supernatant, called the hMSCs CM (i.e., the secretome), was then placed at −80°C until it was required.

### Stereotaxic Surgeries

#### 6‐Hydroxidopamine Lesions

**Figure 1:**
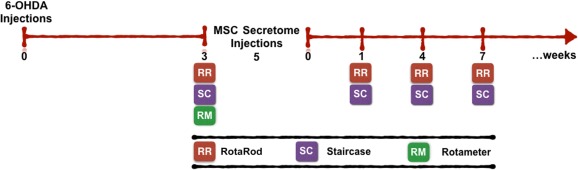
Experimental design. The Parkinson's disease model was induced by a unilateral 6‐OHDA injection into the medial forebrain bundle. Three weeks later, animal behavioral analysis (through RotaRod, staircase, and rotameter tests) was performed to validate the model. Afterward, animals were treated with the human MSC (hMSC) secretome, which was injected in the substantia nigra and striatum, and at 1, 4, and 7 weeks after surgery, and behavioral assessment was performed to address hMSC secretome effects. Abbreviations: MSC, mesenchymal stem cell; 6‐OHDA, 6‐hydroxidopamine.

Ten‐week‐old Wistar‐Han male rats (approximate weight, 300 g; Charles River, Barcelona, Spain, http://www.criver.com/) were housed (2 per cage) and maintained in a controlled environment at 22°C–24°C and 55% humidity, on 12‐hour light/dark cycles and fed with regular rodents’ chow and tap water ad libitum. Animals were handled for 1 week before beginning injections to reduce the stress induced by the surgical procedures. All manipulations were done after consent from the Portuguese National Authority for animal experimentation, Direção Geral de Veterinária (ID: DGV28421), and in accordance with the regulations on animal care and experimentation (European Union Directive 2010/63/EU). Thus, for surgical procedures, under combined ketamine (75 mg/kg) and medetomidine anesthesia (0.5 mg/kg, i.p.), animals (*n* = 36) were placed on a stereotaxic frame (Stoelting, Wood Dale, IL, http://www.stoeltingco.com) and unilaterally injected, using a Hamilton syringe with a 30‐gauge needle (Hamilton, Bonaduz, Switzerland, http://www.hamiltoncompany.com), with either vehicle (sham group, *n* = 11) or 6‐hydroxidopamine (6‐OHDA; *n* = 25; Sigma‐Aldrich) directly into the medial forebrain bundle (MFB) (coordinates related to Bregma: anteroposterior [AP], −4.4 mm; medial lateral [ML], +1.0 mm; dorsoventral [DV], −7.8 mm 1, according to the Paxinos and Watson brain atlas 36). This model was chosen because the literature indicates 6‐OHDA infusions into the MFB can serve as an important tool to study neuroprotective therapies for PD, such as the application of cell grafts or growth factors (e.g., the hMSC secretome) 37, 38, 39. Sham animals received 2 μl of 0.2 mg/ml ascorbic acid in 0.9% of NaCl; 6‐OHDA animals were injected with 2 μl of 6‐OHDA hydrochloride (4 μg/μl) with 0.2 mg/ml ascorbic acid in 0.9% of NaCl at a rate of 1.0 μl/minute. After each injection, the needle was left in place for 4 minutes to avoid any backflow up the needle tract. Three weeks after the surgery, behavioral assessment was performed (Fig. 1).

#### Surgical Treatment: Injection of hMSC CM

Five weeks after the injection of 6‐OHDA, we proceeded to inject the hMSC secretome. After anesthesia was administered, as described in the previous section, animals were placed on a stereotaxic frame and unilaterally injected, using an Hamilton syringe with a 30‐gauge needle, with either the vehicle (Neurobasal‐A medium [Thermo Fisher Scientific Life Sciences] with kanamycin; 6‐OHDA group; *n* = 13), or hMSC CM (6‐OHDA_hMSC CM; *n* = 12) directly in the SNc (coordinates related to Bregma: AP, −5.3 mm; ML, +1.8 mm; DV, −7.4 mm) and into four different striatum (STR) coordinates (AP, −1.3 mm; ML, 4.7 mm; DV, −4.5 mm and −4.0 mm; AP, −0.4 mm; ML, 4.3 mm; DV, −4.5 mm and −4.0 mm; AP, 0.4 mm; ML, −3.1 mm; DV, −4.5 mm and −4.0 mm; AP, 1.3 mm; ML, 2.7 mm; DV, −4.5 mm and −4.0 mm) 36, 40. In the SNc, 6‐OHDA animals received 1 injection of either 4.0 μl of Neurobasal‐A medium with kanamycin or hMSC secretome (as CM), which was injected at a rate of 1.0 μl/minute. In STR, 6‐OHDA animals received either 2.0 μl of Neurobasal‐A medium with kanamycin or hMSC CM in each of the 4 coordinates, making a final volume of 8 μl. After each injection, the needle was left in place for 8 minutes in the SNc, and 4 minutes in the STR to avoid any backflow up the needle tract. At 1, 4, and 7 weeks following surgery, behavioral assessment was performed (Fig. 1).

### Behavioral Assessment

#### RotaRod

Motor coordination and balance of animals (sham, *n* = 11; 6‐OHDA, *n* = 13; 6‐OHDA_hMSC CM, *n* = 12) was evaluated using the TSE RotaRod System (catalog no. 3376‐4R; TSE Systems, Chesterfield, MO, http://www.tse‐systems.com) under an accelerating protocol previously described 41. The first 3 days of testing served as training for the animals. Following this, animals underwent a four‐trial test under an accelerating protocol starting at 4 rpm and reaching 40 rpm in 5 minutes. Animals were allowed to rest for at least 20 minutes between each trial. On day 4, using the same protocol, the animal latency to fall was recorded.

### Skilled Paw‐Reaching Test (Staircase Test)

Skilled paw reaching (also called the staircase test) was performed with double staircase boxes (catalog no. 80300; Campden Instruments, Lafayette, IN, http://www.campdeninstruments.com), as previously described 42. The shape and dimensions of the boxes were similar to the ones described by Montoya et al. 43. The apparatus consists of a clear chamber with a hinged lid that was developed to assess the independent forelimb use in skilled reaching and grasping tasks. A narrow compartment, with a central platform running along its length, is connected to this chamber. The removable double staircase with seven steps on each side can be inserted in the space between the platform and the box walls. Five pellets were placed into each well of the double staircase apparatus. On the first 2 days, the rats (sham, *n* = 11; 6‐OHDA, *n* = 13; 6‐OHDA_hMSC CM, *n* = 12) were familiarized with the test and pellets that were available for 5 and 10 minutes on days 1 and 2, respectively. During the test session, animals were kept inside the box and had 15 minutes to reach, retrieve, and eat food pellets present on the steps. All sessions were performed at the same time of day and with food‐restricted animals. After each test interval, animals were removed from the staircase boxes and the uneaten pellets were counted.

### Apomorphine Turning Behavior

To test apomorphine‐induced turning behavior (rotameter test), animals’ necks were subcutaneously injected with a solution of 0.05 mg/kg apomorphine hydrochloride (Sigma‐Aldrich) dissolved in 1% of ascorbic acid in 0.9% of NaCl, and then placed in metal testing bowls (MED‐RSS, Med Associates, Fairfax, VT, http://www.med‐associates.com) for 45 minutes. The number of contralateral rotations of the bowl was digitally recorded, which allowed assessment of the effects of the injection vehicle (0.2 mg/ml of ascorbic acid in 0.9% of NaCl) and 6‐OHDA (with 0.2 mg/ml of ascorbic acid in 0.9% of NaCl). This test was used to validate the model (after 6‐OHDA injections). Apomorphine is a strong dopamine agonist, and its repeated use could lead to an overstimulation of the dopaminergic system, which could impair the adequate interpretation of the impact of hMSC secretome on the functional outcomes of the animals 44, 45, 46.

### TH Immunohistochemistry

After 13 weeks (including the development of the lesion and consequent treatment), the animals were killed with sodium pentobarbital (Eutasil, 60 mg/kg i.p.; Ceva Saúde Animal, Algés, Portugal, http://www.ceva.pt) and transcardially perfused with 4% paraformaldehyde (Merck, Lisbon, Portugal, http://www.emdgroup.com) in 0.1 M phosphate‐buffered saline (PBS). Striatal and mesencephalon coronal sections, 30 μm thick, were obtained with a vibratome (model no. VT1000S; Leica Biosystems, Wetzlar, Germany, http://www.leicabiosystems.com). Four series of consecutive sections were obtained and one was processed as a free‐floating tyrosine hydroxylase (TH) immunohistochemistry. Sections were immersed for 20 minutes in 1 M PBS with 3% H_2_O_2_, followed by blocking for 2 hours with 5% fetal calf serum (FCS; Thermo Fisher Scientific Life Sciences) in 1 M PBS. Sections then were incubated overnight (at 4°C) with the rabbit anti‐mouse TH primary antibody (1:2,000 [Merck, Billerica, MA, http://www.emdmillipore.com]) in 2% of FCS in 1 M PBS, followed by incubation for 30 minutes with a biotinylated secondary anti‐rabbit antibody, and another 30‐minute incubation with an avidine/biotine complex (Thermo Fisher Scientific Life Sciences). Antigen visualization was performed using 25 mg of 3,3′‐diaminobenzidine tetrahydrochloride (Sigma‐Aldrich) in 50 ml of Tris‐HCl 0.05 M, pH 7.6, with 12.5 μl of H_2_O_2_, and stopped at the desired time. Sections were then mounted on superfrost slides and thionin countercoloration was performed.

To ensure a representative sampling among all the animals when quantifying the SNc TH‐positive cells, six identical TH‐labeled sections spanning the entire mesenphalon were chosen, including all the portions of the SNc. Using a bright‐field microscope (model no. BX51; Olympus, Center Valley, PA, http://www.olympusamerica.com) equipped with a digital camera (PixeLINK PL‐A622, CANIMPEX Enterprises, Halifax, NS, Canada), and with the help of Visiomorph software (V2.12.3.0; Visiopharm, Hørsholm, Denmark, http://www.visiopharm.com), the boundaries of the SNc area were drawn. The delineation of this region was performed through identification of anatomic standard reference points and with the help of a rat brain atlas 36. Samples were analyzed using the Visiomorph software, and total TH^+^ cells in the SNc area were counted in both hemispheres. Data are presented as the percentage of remaining TH^+^ cells in the injected side, compared with control side.

### Striatal Fiber‐Density Measurement

For estimating TH immunoreactive striatal fibers, total immunoreactivity of all TH fibers was measured by densitometry, as described by Febbraro et al. 47. For this purpose, 4 TH‐immunostained prosencephalon sections representing the coordinates of injection sites within the striatum were selected and photographed (×1 objective) with an SZX 16 Microscope fitted to a DP‐71 digital camera (both Olympus). Photos were converted to gray scale using the Image J program, version 1.48 (National Institutes of Health, https://imagej.nih.gov/ij/) and analyzed for gray intensity after calibrating the software program. This was done using the “optical density step tablet” to determine the optical density (O.D.) of the selected sections and performed according to program instructions. This method provided a gross estimation of Parkinsonian pathology on lesioned side. From this, striatum O.D. values were determined in both hemispheres using a 1.1‐mm^2^ rectangular grid, encompassing the injection sites, as determined by anatomical references and rat brain atlas 36. The corpus callosum (internal control) O.D. was also measured in both hemisphere sides to avoid nonspecific background. Thus, TH striatal‐fiber densities were determined by calculating the O.D. difference between the striatum injected with either hMSC CM or Neurobasal‐A medium, and the corpus callosum; as well as between the intact striatum and corpus callosum. The extent of the immunostaining on the lesioned side was expressed as a percentage of the intact side.

### Untargeted Proteomics: Mass Spectrometry Analysis: IDA and Sequential Window Acquisition of All Theoretical Mass Spectra (SWATH) Acquisition

Three biological replicates of hMSC CM were processed as previously described 35. Secreted proteins were precipitated from the concentrated medium using the trichloroacetic acid‐acetone procedure 42. Samples were quantified using the 2D‐Quant Kit (GE Healthcare Biosciences, Pittsburgh, PA, http://www.gelifesciences.com) and 100 μg of protein per sample was subjected to liquid digestion with trypsin (2 μg of trypsin per sample) overnight at 37°C 35, 48, and the formed peptides were desalted using OMIX tips with C18 stationary phase (Agilent Technologies, Glostrup, Denmark, http://www.agilent.com) before liquid chromatography‐tandem mass spectrometry (LC‐MS/MS).

LC‐MS/MS analysis was performed as previously detailed 35, 48 on a Triple TOF 5600 System (SCIEX, Framingham, MA, https://sciex.com) by performing both information‐dependent acquisition (IDA) and SWATH on the same sample. Peptides were resolved by liquid chromatography (nanoLC Ultra 2D; Eksigent, Dublin, CA, http://www.eksigentllc.com) on a Halo Fused‐Core C18 reverse phase column (300 μm × 15 cm, 2.7 μm particles, 90 Å; Eksigent) at 5 μl/minute using an acetonitrile gradient in 0.1% formic acid (2%–35% acetonitrile, in a linear gradient for 25 minutes).

Protein identification from the IDA experiments was obtained by searching against the human and bovine species from UniProt database using the ProteinPilot software (version 4.5; SCIEX). An independent false discovery rate (FDR) analysis using the target‐decoy approach provided with ProteinPilot software was used to assess the quality of the identifications, and positive identifications were considered when identified proteins and peptides reached a 5% local FDR 49, 50.

Additionally, a specific library of precursor masses and fragment ions created from a pool of hMSC samples, described by Teixiera et al. 51, was used to performed protein identification from the SWATH analysis. The chromatographic profiles of the peptides presented in the MSC library were extracted from the SWATH‐mass spectrometry data using the SWATH processing plug‐in for PeakView (version 2.0.01; SCIEX). Peaks were extracted (in an extracted‐ion chromatogram window of 4 minutes) for up to 5 target fragment ions of up to 15 peptides per protein. Positive identification was considered for proteins with at least 1 peptide with a FDR below 1%. Proteins that were identified in a single biological replicate were not considered for further analysis and the final list of hMSC CM‐specific proteins was used for functional characterization of the conditioned medium by Gene Ontology and Pathways analysis using the Protein Analysis Through Evolutionary Relationships (PANTHER) classification system (http://pantherdb.org).

### Bioplex‐Luminex Analysis

The hMSC CM was first concentrated using a 5‐kDa cutoff filter (Vivaspin; GE Healthcare Biosciences) according to manufacturer's guidelines, as previously described 35. Following this, a targeted proteomic analysis for VEGF, nerve growth factor, BDNF, interleukin‐6 (IL‐6), and GDNF was performed using a Bioplex‐Luminex assay. Samples were analyzed in a MAGPIX, Luminex's xMAP instrument (Luminex, Austin, TX, https://www.luminexcorp.com), and each factor concentration was calculated or obtained using Bioplex Manager version 6.1 software (Bio‐Rad Laboratories, Hercules, CA, http://www.bio‐rad.com), and expressed as picograms per milliliter.

### Statistical Analysis

Statistical evaluation of the animal apomorphine behavioral test injection (after 6‐OHDA) was performed using Student's *t* test. For RotaRod and staircase tests, upon 6‐OHDA and hMSC CM or Neurobasal‐A medium injections, analysis of variance repeated measures followed by post hoc Bonferroni for multiple comparisons was performed using the SPSS statistics program (version 22; IBM, Armonk, NY, http://www.ibm.com). Data are presented as mean ± SEM. The significance value was set at *p* < .05.

## Results

### Phenotypic Characterization of 6‐OHDA Lesions

**Figure 2:**
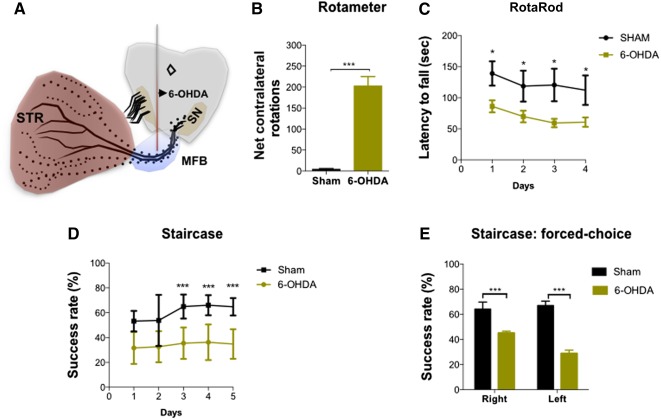
Behavioral characterization of 6‐OHDA induced lesions. **(A, B):** After 3 weeks, the injection of 6‐OHDA in the MFB **(A)** led to an intense turning behavior in the apomorphine‐induced turning behavior when compared with sham group **(B)**. 6‐OHDA‐injected animals also exhibited significant impairment in motor coordination on the RotaRod test **(C)** and in the paw‐reaching test performance **(D, E)**. For rotameter testing, *n* = 11 for the sham group and *n* = 21 for the 6‐OHDA group. For RotaRod testing, *n* = 9 for the sham group and *n* = 17 for the 6‐OHDA group. For the staircase test, *n* = 10 for the sham group and *n* = 21 for the 6‐OHDA group. Data are presented as mean ± SEM. ∗, *p* < .05, ∗∗∗, *p* < .001. Abbreviations: MFB, medial forebrain bundle; 6‐OHDA, 6‐hydroxidopamine. sec, seconds; STR, striatum.

To further evaluate the functional integrity of the animal dopaminergic system after 6‐OHDA injection (Fig. 2A), the apomorphine‐induced turning test was performed at the end of the behavioral assessment. Three weeks after the 6‐OHDA injections, we observed that there was a significantly higher number of apomorphine‐induced turning rotations in the 6‐OHDA‐injected animals when compared with the sham group (*t* = 5.898; *p* < .001; Fig. 2B). Assessment of motor performance also revealed deficits after the 6‐OHDA injections. Motor coordination, as assessed by the RotaRod test, was observed to be impaired in 6‐OHDA‐injected animals (F_(1,24)_ = 16.72; *p* < .001; η^2^_partial_ = 0.411; Fig. 2C). Moreover, in the staircase test (used to assess the forelimb use and skilled motor function), we also observed that the 6‐OHDA‐injected animals were clearly affected, as indicated by a comparison with the animals in the sham group (F_(1,29)_ = 50.81; *p* < .001; η^2^_partial_ = 0.637; Fig. 2D). Finally, in a staircase forced‐choice task (in which animals were forced to choose one of the two staircases), the 6‐OHDA‐injected animals were found to be significantly impaired compared with the those in the sham group (right‐side *t* = 6.66; left‐side *t* = 6.81; *p* < .001; Fig. 2E).

### Transplantation of hMSC CM‐Attenuated Motor Deficits of 6‐OHDA‐Injected Animals

To evaluate the effects of the hMSC CM (i.e., the secretome) in 6‐OHDA‐injected animals, animal motor performances were assessed at 1, 4, and 7 weeks after CM injection during the RotaRod and staircase tests.

**Figure 3:**
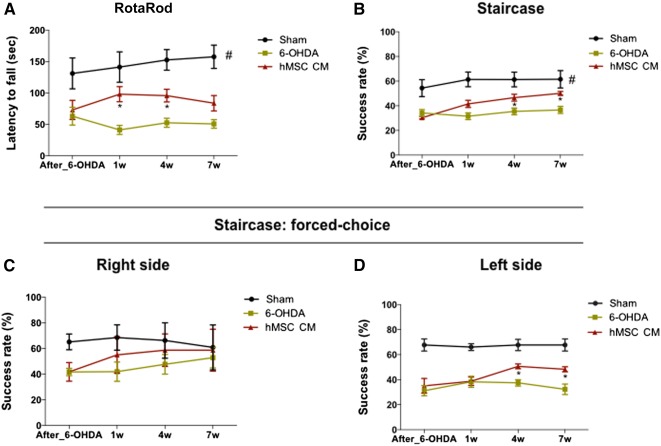
Motor coordination performance after hMSC CM (i.e., secretome) injection into the substantia nigra and striatum. **(A):** Latency to fall was measured in the accelerating RotaRod test, demonstrating that the hMSC CM‐injected animals had a significant improvement (at 1 and 4 weeks after injection; *p* < .05) in their motor coordination when compared with the 6‐OHDA group 6‐OHDA. **(B):** Paw‐reaching performance (assessed by the staircase test) also demonstrated a significant improvement (at 4 and 7 weeks after injection; *p* < .05) of the forelimb coordination of the hMSC CM‐injected animals when compared with the 6‐OHDA group. Even when the animals were submitted to a paw‐reaching forced‐performance task, the hMSC CM‐injected animals presented a better performance on the affected side (at 4 and 7 weeks after injection; *p* < .05) when compared with the 6‐OHDA group **(C, D)**. For the RotaRod test, the numbers tested in the sham, 6‐OHDA, and hMSC CM groups were 9, 9, and 8, respectively. For staircase test, the numbers tested in the sham, 6‐OHDA, and hMSC CM groups were 10, 10, and 11, respectively. Data are presented as mean ± SEM. ∗, *p* < .05; #, sham animals statistically different from 6‐OHDA‐ and hMSC CM‐injected animals, *p* < .001. Abbreviations: hMSC CM, human mesenchymal stem cell‐conditioned medium; 6‐OHDA, 6‐hydroxidopamine; sec, seconds; w, weeks.

For the RotaRod test, after CM injection, statistical analysis showed an effect for the factor treatment (F_(2,23)_ = 31.58; *p* < .001; η^2^_partial_ = 0.733) but no effect for the factor time (F_(1.82, 41.89)_ = 0.727; *p* = .477; η^2^_partial_ = 0.031) and no interaction between these factors (F_(3.64, 41.89)_ = 2.24; *p* = .087; η^2^_partial_ = 0.163). Comparing the CM‐injected animals with the untreated group (i.e., the 6‐OHDA group), post hoc testing revealed that the motor coordination of the former group significantly improved after hMSC CM injection (*p* = .016; Fig. 3A). The same improvements were also observed with the staircase test, which was used to address the forelimb use and for which the success rate of eaten pellets was evaluated. After the CM injection, statistical analysis revealed a significant effect for the treatment (F_(2,28)_ = 48.33; *p* < .001; η^2^_partial_ = 0.775), for the factor time (F_(2.57, 71.96)_ = 18.01; *p* < .001; η^2^_partial_ = 0.391), and the interaction between these factors (F_(5.14, 71.96)_ = 6.17; *p* < .001; η^2^_partial_ = 0.306). Comparing the CM‐injected animals with the 6‐OHDA group, post hoc analysis revealed that the injection of hMSC CM led to a significant improvement of the success rate of eaten pellets (*p* = .018; Fig. 3B). In addition, in the forced‐choice task, the hMSC CM‐injected animals also had improved performance. Statistical analysis revealed an effect for the factor treatment (F_(2,28)_ = 34.06; *p* < .001; η^2^_partial_ = 0.709), the factor time (F_(2.87, 80.35)_ = 3.22; *p* = .029; η^2^_partial_ = 0.103), and the interaction between these factors (F_(5.74, 80.35)_ = 3.13; *p* = .009; η^2^_partial_ = 0.183). Post hoc testing of the effects of the hMSCs CM revealed an increased success rate of eaten pellets (in the affected side) when compared with the 6‐OHDA group (left side, *p* < .05, Fig. 3C; right side, *p* = .438, Fig. 3D).

### Transplantation of the hMSC Secretome Restored the Neuronal Structure

**Figure 4:**
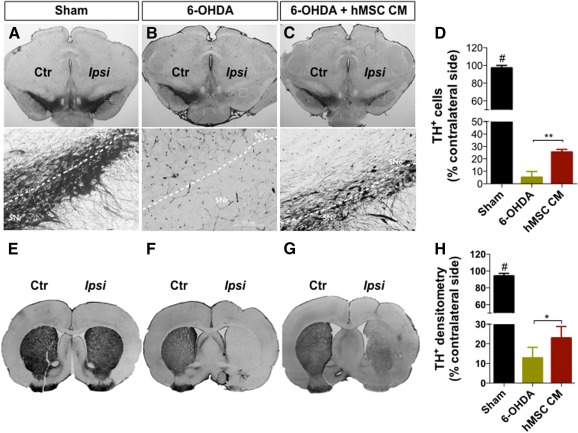
Representative photomicrographs of brain slices stained for TH. Compared with the sham group **(A, E)**, all animals that were submitted to 6‐OHDA injection exhibited reduced TH staining both in the SNc and striatum (STR). However, animals injected with the hMSC CM (i.e., secretome) **(C, G)** had significantly more TH‐positive staining when compared with the 6‐OHDA group **(B, F)**. **(D):** Quantification of TH‐positive cells on SNc revealed approximately 95% degeneration in the 6‐OHDA group and approximately 75% in the CM‐injected animals. **(H):** At the STR level, the quantification of TH staining revealed a degeneration of approximately 88% in the 6‐OHDA group, and approximately 77% in the CM‐injected animals. Data are presented as mean ± SEM. There were nine animals in the sham group and in the 6‐OHDA group, and eight in the hMSC CM group. ∗, *p* < .05, ∗∗, *p* < .01; #, sham animals statistically different from 6‐OHDA‐ and hMSCs CM‐injected animals, *p* < .001. Scale bars = SNc, 200 μm; STR, 1 mm. Abbreviations: Ctr, control; hMSC CM, human mesenchymal stem cell‐conditioned medium; Ipsi, ipsilateral; 6‐OHDA, 6‐hydroxidopamine; SNc, substantia nigra pars compacta; SNr, substantia nigra pars reticulata; TH+, tyrosine hydroxylase positive.

To further analyze the effects of the 6‐OHDA injections and the resulting treatment with hMSC CM, histological analyses for TH were performed. We found that there was a significant loss of DA neurons after injection of 6‐OHDA into the SNc (Fig. 4A–4C). Indeed, statistical analysis revealed an effect for the treatment (F_(2, 23)_ = 213.34; *p* < .001; η^2^_partial_ = 0.949). Injection of the hMSC CM appeared to play a role in the survival of DA neurons: There was a significantly higher number of TH‐positive cells observed in the SNc (CM: 25.36% ± 5.45%) when compared with the 6‐OHDA group (5.08% ± 1.60%) (*p* < .01; Fig. 4D). The same tendency was also observed in the striatum (Fig. 4E–4G) by assessing TH‐positive fibers by densitometry analysis. Statistical analysis revealed results from the treatment (F_(2, 23)_ = 350.69; *p* < .001; η^2^_partial_ = 0.997) in which the injection of hMSC CM was able to increase the TH expression levels when compared with the 6‐OHDA group (*p* < .05; Fig. 4H).

### hMSCs Secretome: A Source of Neuroregulatory Molecules for PD

**Figure 5:**
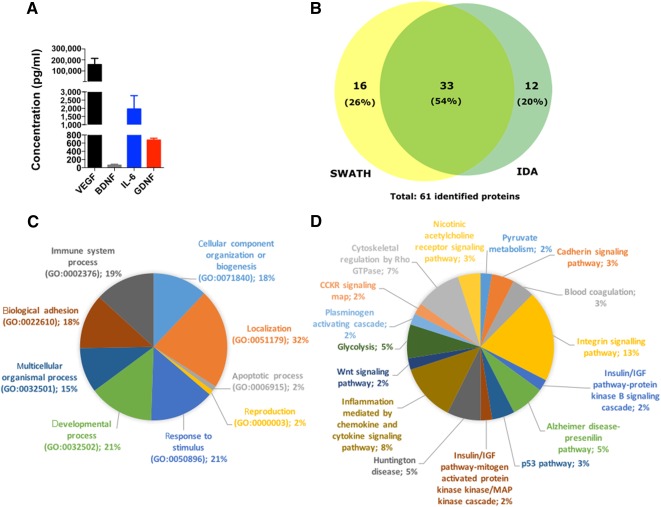
The hMSC secretome as a source of neuroregulatory molecules for PD. **(A):** Through Bioplex‐Luminex‐based analysis, we were able to observe an active secretion of neurotrophic factors such as VEGF (highly expressed; *p* < .05), BDNF, IL‐6, and GDNF—important regulators/modulators on dopaminergic neuronal survival and protection. Through a combined MS analysis **(B)**, we also found 61 proteins secreted by hMSCs that, in accordance with Gene Ontology analysis of biological processes **(C)** and Protein Analysis Through Evolutionary Relationships (PANTHER) pathways **(D)** have neuroregulatory properties such as cystatin C, glia‐derived nexin, galectin‐1, and pigment epithelium‐derived factor (PEDF) **(B)**. Like the other neurotrophic factors, PEDF was found to be a neuroprotective molecule of the dopaminergic system. Data are presented as mean ± SEM; *n* = 3. Abbreviations: BDNF, brain‐derived neurotrophic factor; GDNF, glial cell line‐derived neurotrophic factor; IDA, information‐dependent acquisition; IGF, insulin‐like growth factor; IL‐6, interleukin‐6; SWATH, Sequential Window Acquisition of All Theoretical Mass Spectra; VEGF, vascular endothelial growth factor.

To identify key molecules in the hMSC secretome that were eliciting the favorable behavioral responses in the 6‐OHDA‐treated animals, we characterized the secretome through targeted and nontargeted proteomic approach‐based analyses (i.e., Bioplex assays and a combined mass spectrometry approach. From the Bioplex analyses, we were able to observe that hMSCs secreted important neurotrophic factors such as VEGF, BDNF, IL‐6, and GDNF (Fig. 5A). In addition to these findings, and through the combined MS analysis (Fig. 5B), we also found additional proteins (according to the UniProtKB/Swiss‐Prot classification) with important actions (Fig. 5C, 5D), including CNS regulators such as Cys C (P01034), glia‐derived nexin (GDN; P07093), galectin‐1 (Gal‐1; P09382), and pigment epithelium‐derived factor (PEDF; P36955) (Table 1).

**Table 1:** Combined list of reproducible proteins as identified by IDA and SWATH proteomic screenings

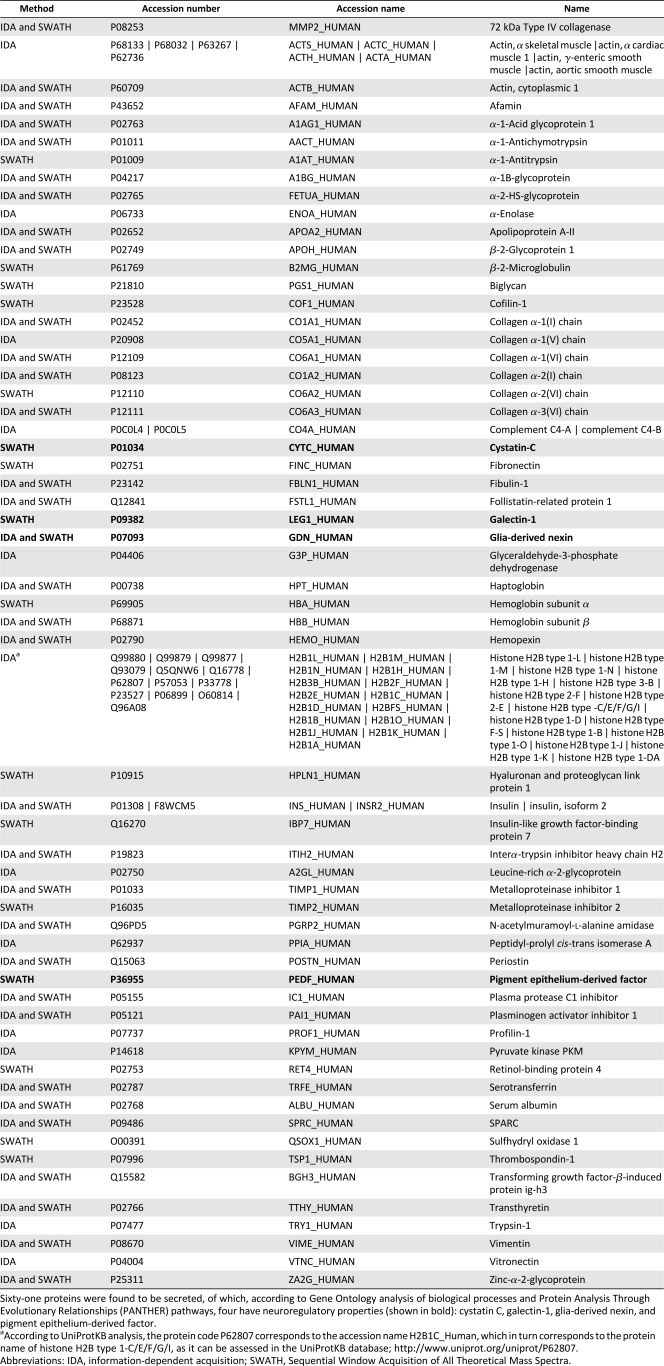

## Discussion

In this study, we used a rat model of PD, based on a unilateral injection of 6‐OHDA into the MFB (Fig. 2A) 1, 41. This model is characterized by a progressive degeneration of DA neurons, leading to the appearance of the main motor symptoms of the disease 1, 52. In the apomorphine‐turning behavior (Fig. 2B), 6‐OHDA‐injected animals displayed an intense turning behavior (*p* < .001) when compared with the sham group, indicating a decline in the functional integrity of the DA system. In addition, we have also verified that the animals’ motor coordination was affected (Fig. 2C, 2D), which correlated with previous reports showing impaired coordination and skilled motor function in animals with DA lesions 41, 53.

Considering the effects of the hMSC secretome (i.e., the CM) on motor coordination (as seen in the RotaRod test), we observed that its injection was able to improve the motor performance of the CM‐injected animals when compared with the 6‐OHDA group (Fig. 3A). Interestingly, despite these improvements, we also observed that the effects of the hMSC secretome declined over the time, probably through the in situ consumption of the factors contained in it. Therefore, future studies should investigate the temporal effects of hMSC secreted factors.

In the staircase test, which assessed the paw‐reaching motor function, we observed that hMSC CM injection improved the success rate of eaten pellets in the CM‐injected animals when compared with the 6‐OHDA group (Fig. 3B). Moreover, in the forced‐choice task, the injection of hMSC CM was observed to be an enhancer (on the affected side) of the paw‐reaching motor performance when compared with the 6‐OHDA group (Fig. 3D). Moreover, we also observed that the administration of the secretome induced an increase on the TH^+^ neurons and fibers (Fig. 4), which most likely mediated the positive functional improvements we observed. Although, to our knowledge, no reports have been presented with an hMSC secretome in PD animal models to date, similar results were observed by Cova et al. 28 and Sadan et al. 33 for another approach whereby MSC cells were transplanted. These authors correlated animal improvements with the increase in the local expression of BDNF and GDNF by transplanted MSCs. Recently, Cerri et al. 54, also supporting the secretome theory, demonstrated that after transplantation of MSCs, functional balance of the dopaminergic system was restored; they attributed these outcomes to an in situ secretion of BDNF by MSCs.

To further understand which factors present in the hMSCs secretome could be involved in this phenomena, nontargeted and targeted proteomic approaches were performed. The latter revealed that molecules such as VEGF, BDNF, IL‐6, and GDNF (Fig. 5A), described as stronger modulators of dopaminergic survival and protection, were present in the hMSC secretome 55, 56, 57, 58. Studies have described VEGF as a promoter of neuroprotection of DA neurons, by stimulating the neuropilin receptor expressed on DA neurons 59 or through the activation of glial cells and by promoting angiogenesis 60. BDNF has also been described as a credible protective molecule in the degenerative process of PD 55. In fact, when applied in vitro, BDNF induced the differentiation and neurite outgrowth in DA neurons 61. Indeed, in an organotypic model of PD, BDNF was also capable of protecting DA neurons from 6‐OHDA treatment, even when applied after the addition of toxin 62. In vivo, in nonhuman primates, BDNF has demonstrated the ability to reduce DA neuronal loss, suggesting a significant protective effect 63. Such evidence reinforces the importance of BDNF in PD; from the molecular point of view, it has been suggested that the downregulation of BDNF expression in the SNc might be one of the earlier steps at the onset of PD, which leads to an increased sensitization of DA neurons 64. Secretion of IL‐6 by MSCs has been reported to play important roles in scavenging superoxide radicals by increasing the antioxidant enzyme activity, through signal transducers and activators of transcription pathways, leading to the protection of neuronal cells 56. Finally, the therapeutic effects of GDNF have largely been assessed in preclinical and clinical models of PD, leading to protective effects and motor performance amelioration 55, 65, 66. Indeed, in vitro studies have suggested that these protective effects of GDNF on DA neurons involves the activation of the mitogen‐activated protein kinase (MAPK) and phosphoinositide 3‐kinase (PI3K) intracellular pathways 67. In vivo, it has been demonstrated that the neuroprotective effects of GDNF were mediated by its capacity to inhibit proapoptotic molecules such as JNK and p38, and activate prosurvival Akt and MAPK pathways 68. In addition to these neuroprotective effects, GDNF was also described as an antioxidant agent because it was found to be able to enhance the activity of enzymes involved in the detoxification of reactive oxygen species such as superoxide dismutase, catalase, and glutathione peroxidase, respectively 69.

However, in addition to these well‐known neurotrophic factors, we also found that hMSCs produced additional molecules (Fig. 5B) with neuroregulatory potential, such as Cys C, GDN, Gal‐1, and PEDF, which have been reported to have important actions in the migration, differentiation, and neuroprotection mechanisms both in vitro and in vivo 70, 71, 72, 73. Of these, only PEDF was found to be an important neurotrophic and neuroprotective molecule in the context of PD 73, 74. This has been confirmed by Falk et al. 74, who, when comparing other factors (e.g., GDNF family) used in the treatment of PD, stated that PEDF has advantages in the ease of delivery and functional outcomes. Indeed, recent reports have suggested that upregulation of PEDF occurs in response to acute insults in the dopaminergic system, suggesting that PEDF may act as an endogenous neuroprotective molecule, triggering neuronal survival and behavioral improvements in animal models of PD 74, 75. These neuroprotective actions have been correlated by the fact that PEDF is able to stimulate the activation of the NF‐κB signaling cascade, which allows NF‐κB to act as a transcription factor that induces the expression of factors critical to neuronal protection and survival, including BDNF and GDNF 74. Thus, we hypothesize that the modulating effect on DA neurons triggered by the hMSCs secretome could be related to the increased presence or expression of VEGF, BDNF, IL‐6, GDNF, and PEDF. Our findings suggest that this stimulation by the hMSC secretome is not dependent on the presence of just one secreted factor but several, as noted by our targeted and nontargeted proteomic analyses.

## Conclusion

After the injection of an enhanced bioreactor‐produced hMSC secretome (i.e., CM), we observed an improvement in animal behavior when compared with the 6‐OHDA group. We also found that the injection of the hMSC secretome was able to increase the densities of TH‐positive cells, a fact that most likely explains the improved behavioral performance of the CM‐injected animals. In addition to the important neuroregulatory molecules present in the hMSC CM (e.g., VEGF, BDNF, IL‐6, and GDNF), the presence of PEDF might also have a role in its outcomes because it has been described as an important neurotrophic and neuroprotective molecule in PD. Overall, our results suggest strongly that the use of the hMSC secretome may be a new and important tool for the treatment of PD because the secretome is able to modulate the DA neuronal survival and animal behavior.

## Author Contributions

F.G.T.: conception and design, collection and/or assembly of data, data analysis and interpretation, manuscript writing; M.M.C. and A.J.R.: data analysis and interpretation; K.M.P.: data analysis and interpretation, manuscript writing; B.M.‐P.: collection and/or assembly of data; S.A. and B.M.: collection and/or assembly of data, data analysis and interpretation; L.A.B.: provision of study material, data analysis and interpretation, manuscript writing; N.S.: data analysis and interpretation, manuscript writing, final approval of manuscript; A.J.S.: financial support, provision of study material, data analysis and interpretation, manuscript writing, final approval of manuscript.

## Disclosure of Potential Conflicts of Interest

The authors indicated no potential conflicts of interest.
